# Paracrine signals of mesenchymal stem cells induce epithelial to mesenchymal transition in gastric cancer cells 

**Published:** 2019

**Authors:** Parisa Azizi, Sogol Mazhari, Samaneh Tokhanbigli, Fatemeh Naderi Noukabadi, Elahe Daskar Abkenar, Esmaeil Shamsafzali, Kaveh Baghaei

**Affiliations:** 1 *Basic and Molecular Epidemiology of Gastrointestinal Disorders Research Center, Research Institute for Gastroenterology and Liver Diseases, Shahid Beheshti University of Medical Sciences, Tehran, Iran*; 2 *Gastroenterology and Liver Diseases Research center, Research Institute for Gastroenterology and Liver Diseases, Shahid Beheshti University of Medical Sciences, Tehran, Iran *; © equal contributed

**Keywords:** Gastric Cancer, Epithelial mesenchymal transition, Mesenchymal stem cells

## Abstract

**Aim:**

Given the high similarity of phenotypical and secretory properties of mesenchymal stem cells and fibroblasts, this study investigated the possibility of inducing EMT process by mesenchymal stem cells.

**Background:**

Annually, more than 13% of deaths worldwide occur due to cancer. One of the main reasons for the high mortality rate is due to the metastasis of cancer stem cells. Induction of metastasis occurs during the EMT process, which can also be stimulated by fibroblast cells.

**Methods:**

Mesenchymal stem cells (MSCs) were isolated and sub-cultured until passage 3 or 4. AGS cells were co-cultured with MSCs for 4 days. As the positive control group, AGS cells were treated with TGF-β (10ng/ml) for 48h. Finally, the mRNA expression level of Vimentin, β-catenin, Snail, and E-cadherin as the EMT pattern, were evaluated by RT-PCR technique.

**Results:**

Our findings indicated that AGS cells’ crosstalk with MSCs significantly upregulated fibroblast markers including Vimentin and Snail expression. However, no significant changes were identified for β-catenin gene expression. Additionally, AGS treatment with MSCs resulted in diminished E-cadherin in the targeted cells.

**Conclusion:**

Based on the results, the AGS cells crosstalk with MSCs activates induction of epithelial mesenchymal transition, which is confirmed through the elevation of Vimentin and Snail expression and reduction of E-cadherin expression as a specific epithelial marker. However, it seems that MSc was not effective on Wnt/ β-catenin signal gastric cancer cell line.

## Introduction

 Gastric cancer is a multifactorial process recognized as one of the leading causes of cancer deaths worldwide (1). Gastric cancer is the second and fourth most common cancer in men and women, respectively. One of the major causes of the high mortality rate of this cancer is associated with metastasis and spreading out to other organs including the liver, lung, bone, and lymph nodes (2). Numerous studies have shown a direct relationship between metastasis and the phenomenon of Epithelial-mesenchymal transition (EMT)(3). EMT is a conserved evolutionary process, in which epithelial cells transform into fibroblast phenotype and express mesenchymal markers (4). During this process, epithelial cells lose the potential of cell-cell adhesion and cell polarity and acquire the migration phenotype and invasion properties by reducing the expression of epithelial markers such as E-cadherin and enhancing the expression of mesenchymal markers such as Vimentin (5). Furthermore, EMT is associated with a variety of signaling pathways including Transforming Growth Factor-β (TGF-β), Platelet Derived Growth Factor (PDGF), and Hepatocyte Growth Factor (HGF), Notch, and RAS (6).

Numerical researches has indicated that cancer cells during EMT show cancer stem cells properties and their gene expression profile is approximately similar to the cancer stem cells (7). Thus, the epithelial cells that are exposed to EMT either by TGF-β treatment or by inducing the expression of transcriptional inhibitors such as E-cadherin show higher CD44^high^ and CD24^low^ cells (8). 

Fibroblasts have been shown to have a strong ability in induction of EMT in cancer cells by secretion of TGF-β and IL-6 cytokine (9). This pathway results in elevation of Snail, Vimentin, and β-catenin gene expression as well as reducing the E-cadherin gene expression in targeted cells (5). On the other hand, mesenchymal stem cells (MSCs) are a heterogeneous subset of adult stem cells, which can be easily aspirated from every postnatal connective tissue. Along with MSCs self-renewing, differentiating, and anti-inflammatory properties, varied clinical trials have utilized MSCs for regenerative medicine applications (10). Additionally, numerous studies have demonstrated the therapeutic impact of MSCs-derived conditioned medium on amelioration of injuries and autoimmune diseases (11). However, the results of MSc therapy on cancer disease are controversial. It has been shown that MSC has a double edge role in cancer therapy (12). Some studies indicated the positive and inhibitory impacts of MSCs on tumor growth (13). However, recent studies have shown conflicting results regarding the stimulatory effects of MSCs on tumor pathogenesis through supporting tumor microenvironments and stimulation of tumor growth ((14).

 This inappropriate function can be justified by fibroblast-like properties of MSc. MSCs have flat elongated/spindle phenotypes similar to fibroblasts (15). Further, secretory factors of mesenchymal cells and fibroblasts are highly similar. These two cell types also express the same identical cell surface markers pattern including CD166(+) , CD133(-), CD105(+), CD90(+), CD73(+), CD71(+), CD45(-), CD44(+), CD34(-), CD31(-), CD29(+), and CD14(-) (16).

 Regarding similarity of phenotype and cytokine panel between MSC and fibroblasts, exerting similar effects on cancer cells is not out unexpected. In the present study, we considered the paracrine effects of mesenchymal stem cells on gastric cancer cell line regarding EMT. 

## Methods

**Cell culture**

**MSC isolation and expansion**

Human bone marrow mesenchymal stem cells were isolated from iliac crest biopsies based on a protocol previously described by the authors with a few modifications (17). In summary, the bone marrow was gathered into an EDTA tube, then buffy coat layer was segregated through centrifugation (450 xg, 10 min). In order to achieve MSCs, the isolated buffy coat with an equal volume of Ficoll was centrifuged (400 xg, 20 min). The harvested cells were expanded in a monolayer culture and maintained in complete medium of Dulbecco Modified Eagle’s Medium (DMEM, Gibco-Invitrogen, USA), containing 15% Fetal Bovine Serum (FBS, Gibco-Invitrogen, USA) and 1% penicillin/ Streptomycin (Gibco-Invitrogen, USA) at 37 °C and in a humidified atmosphere containing 5% CO2. The bone marrow mesenchymal stem cells at passages 4-6 were used for the experiments in this study. These cells were shown to be positive for CD90, CD73, CD105, and negative for CD14, CD34, and CD45 (all eBioscience, USA) flow-cytometry analysis, and were capable of osteoblastic and adipogenic differentiation based on established protocols (data not shown).

**Gastric cancer cell line**

Human gastric cancer cell line (AGS) was obtained from Research Institute of Gastroentrology and Liver Diseases (Shahid Beheshti Medical University, Iran). It was cultured under exponential growth in complete Roswell Park Memorial Institute medium (RPMI) (Gibco-Invitrogen, USA) supplemented with 15% FBS, 1% penicillin /streptomycin, 1% non-essential amino acids (Gibco-Invitrogen, USA), and 1% L-glutamine (Gibco-Invitrogen, USA), and cultured in a 5% humidified CO2 incubator at 37°C.

**Induction of Epithelial-Mesenchymal Transition by AGS-MSCs co-culture system**

Once the cells reached the appropriate confluency, the MSCs and AGS cells were trypsinized (Trypsin-EDTA, Gibco, USA), then counted for transformation to a co-culture insert plate 0.4 µm (Lifescience, USA). For this purpose, approximately 2×10^5^ AGS cells in complete RPMI medium supplemented with 10% FBS and 1% penicillin /streptomycin were added to the basal surface of each insert plate. Next, 150000 MSCs in DMEM medium, containing 10% FBS, and 1% penicillin/ streptomycin were transported to the upper surface of each well. Eventually, after 3 days, the AGS cell were detached by Trypsin-EDTA and utilized for subsequent tests.

**Table 1:** The sequence of primers used for EMT markers detection

Accession number	Forward	Gene
NG_013302.2	FW: GGGTAGGGTAAATCAGTAAGAGGT	β-catenin
	RV: GCATCGTATCACAGCAGGTT	
NG_008021.1	FW: TGCTCTTGCTGTTTCTTCGG	E-cadherin
	RV: CTTCTCCGCCTCCTTCTTC	
NG_005985.4	FW: CACTATGCCGCGCTCTTTC	Snail
	RV: TGCTGGAAGGTAAACTCTGGAT	
NG_012413.1	FW: CCAGGCAAAGCAGGAGTC	Vimentin
	RV: CGAAGGTGACGAGCCATT	
NG_007073.2	FW: GAAGGTGAAGGTCGGAGTCA	GAPDH
	RV: AATGAAGGGGTCATTGATCA	

**Figure 1:**
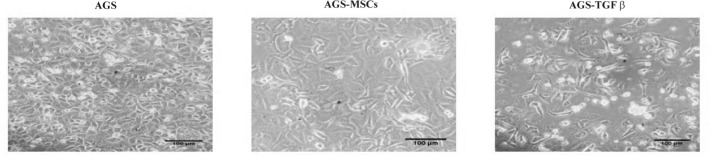
Morphological changes of AGS cells treated with MSCs and TGF-β observed with an inverted microscope ×200 magnification

**Figure 2:**
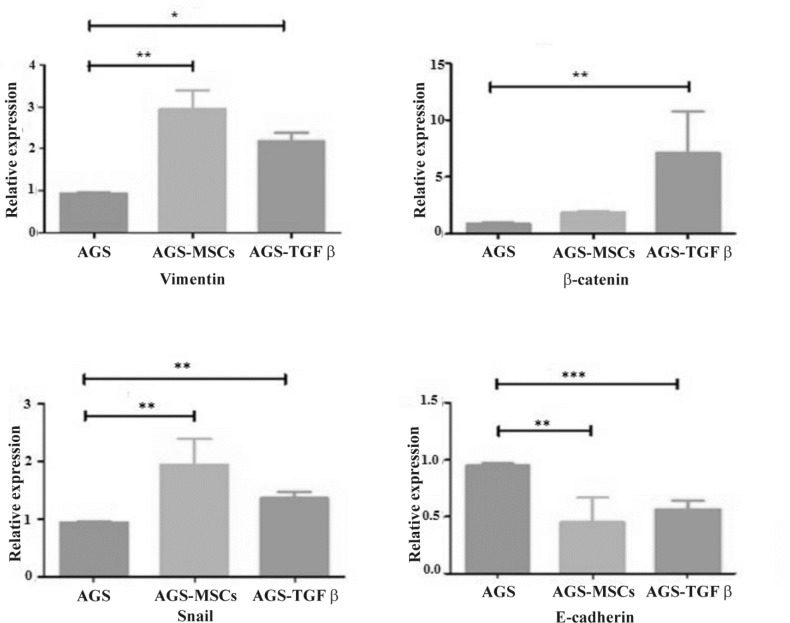
Alteration in the expression level of Vimentin, β-catenin, Snail, and E-cadherin of AGS-MSCs treated group and TGF-β treatment group compared to the AGS without any treatment group. The expression of specific EMT markers were normalized by GAPDH as a housekeeping gene. Bars are indicating the mean± SEM of three times experiments. Independent student t-test was used for statistical analysis. ,* P<0.01, ** P<0.001, and ***P<0.0001 in comparison with the control group

**Treatment of AGS by TGF-β for inducing Epithelial-Mesenchymal Transition**

AGS cells treated with TGF-β1 were used as the control group to be compared with the AGS-MSCs co-culture system. AGS cells were seeded almost 2×10^5^ per well, in 6-well plates in an RPMI medium supplemented with 10% FBS and 1% penicillin /streptomycin and incubated at 37 °C with 5% CO2 for 24 hours. Subsequently, to induce cell differentiation, cell growth decreased by reducing FBS to 0.5% as serum starvation. Simultaneously, 10 ng/ml of TGF-β was added to the culture media. After 48 hours of treating with TGF-β (10ng/ml), the cells were collected and used for the following tests.

**RNA extraction and cDNA synthesis**

Total RNA was isolated by RNA Purification Mini Kit (FavorGen, Taiwan), based on the manufacturer's instructions. The concentration of the total RNA was determined via the Nanodrop (Thermo Fisher Scientific, USA) by absorbance at 260 nm. Subsequently, the extracted RNA was converted to cDNA, according to RevertAid First Strand cDNA Synthesis Kit (Thermo Scientific, USA) using a random hexamers primer in the presence of RNase inhibitor. The quality and concentration of cDNA were measured by Nanodrop Device. The synthesized cDNA was stored at -70 °C until further analysis.

**Quantitative real-time PCR**

To perform quantitative real-time PCR reaction, initially, the primers of the desired genes for assessment of EMT induction (Vimentin, Snail, β-catenin, and E-cadherin) and internal control gene (GAPDH) were designed by primer 3 software and blasted at NCBI. For validation of the accuracy and specificity of the primers, Gene Runner (ver. 6.0.04) was used. The sequence of the primers is shown in Table 1. In the next step, the synthesized cDNA was amplified using a SYBR Green I PCR Master Mix based on the manufacturer's instructions. A total of 10µl SYBER Master Mix, 0.8 µl Forward primer, 0.8 µl Reverse primer, 150 ng/µl cDNA as well as dH2O in the final volume 20 µl were used for Real-time PCR reaction. Quantitative analysis of the gene expression level was normalized with GAPDH as the internal control. The alteration in mRNA expression’s level of the target genes was compared with the AGS without any treatment group. Data were analyzed by REST 2009 software version 2.0.13.

**Statistical analysis**

Prism software statistical package version 5 (GraphPad Software, USA) was utilized for data analysis. Independent t-test was applied for examining the differences between the experimental groups with the data presented as mean (SEM). Significance was set as the difference P-value is less than 0.05

## Results

**Phenotypical assessment**

Once treated with MSCs for 3 days, the morphological changes of AGS cells were observed with an inverted microscope (Olympus, Japan). The cells have a plating efficiency of approximately 80% in the medium at passage number 3. The AGS- MSCs treated cells had remarkable phenotypic differences from the AGS cells without any treatment as the control group (Figure 1). Compared to the control group, the majority of AGS cells co-cultured with MSCs obviously gained a fibroblast spindle shape, while the control group had a typical round epithelial cell morphology. **AGS-MSCs crosstalk affects the specific **makers of EMT.

To investigate the impact of MSCs-secretions on induction of EMT, qRT-PCR was performed to detect the expression of Vimentin, β-catenin, Snail, and E-cadherin on targeted cells. The AGS cells without any treatment were used as negative control group while the AGS cells treated with TGF-β were utilized as positive control group. AGS-MSCs crosstalk considerably elevated the expression of EMT markers gene, including Vimentin, β-catenin, and Snail. The expressions of Vimentin and Snail genes were significantly upregulated when AGS cells were co-cultured with MSCs and TGF-β treated groups in comparison with the control group without any treatment (Fig 2 A and C). Based on the results, the co-culture of AGS with MSCs showed more upregulation of Vimentin and Snail in comparison with AGS-TGF-β group. However, there was no significant alteration in the expression of the β-catenin gene in AGS-MSC treated group (Figure 2 B). On the other hand, diminished mRNA level of E-cadherin was observed in both treatment groups without any significant difference (Figure 2 D). AGS-MSCs treated group, similar to TGF-β treated group, induced EMT pattern. The qRT-PCR analysis was obtained from three independent experimental repetitions. The results indicate that mRNA expression levels of Vimentin, β-catenin, and Snail were upregulated in both AGS-MSCs and AGS treated with TGF-β groups compared with the negative control group. On the other hand, the expression level of E-cadherin as a specific marker for epithelial cells was reduced in both treated groups. Hence, the AGS-MSCs treated group has particularly the potential of inducing EMT in AGC cell line.

## Discussion

MSCs are one of the main subsets of adult stem cells, which can be separated from varied tissues such as adipose tissue, bone marrow, and cord blood. Numerous studies have reported that MSCs have unique characteristics such as high proliferation and differentiation capacity, ease of access and separation, and potential of secretion of various components including growth factors and cytokines (18). Hence, MSCs have attracted attention as a promising candidate in various diseases. Taking into account the importance of MSCs, many clinical trials have been performed utilizing mesenchymal stem cells as a therapeutic agent for autoimmune diseases such as diabetes, rheumatoid arthritis, Crohn's disease, lupus erythematosus, and multiple sclerosis (7). Nevertheless, it should be noted that MSCs studies on the tumor pathogenesis have reported controversial results. Numerous studies suggest that MSCs have inhibitory effects on tumor progression through suppressing immune responses, inhibiting angiogenesis, and Akt and Wnt signaling pathways (19–22). Further, some studies have demonstrated that MSCs can induce apoptosis or cell cycle arrest in the G0-G1 phase (23). In contrast to anti-tumor impacts of MSCs, various studies have shown that MSCs-secreted factors including, TWIST, MMP, WNT5A, and TGF-β stimulate tumor growth, progression, and metastasis (24). In the current study, to clarify the interaction between cancer cells and MSCs, we evaluated the potential of EMT induction in AGS cell line co-cultured with MSCs. The qRT-PCR was performed for evaluating EMT-related gene expression pattern, including Vimentin, Snail, β-catenin, and E-cadherin. The expression levels of some transcription factors and protein such as Vimentin, Snail, and β-catenin were upregulated during EMT process causing direct inhibition of epithelial binding mediators, and most importantly E-cadherin (25). Indeed, it is suggested that the interaction between these agents and signaling pathways modulate the EMT process. According to our results, the expression levels of Vimentin and Snail were significantly upregulated as the marker of transition to mesenchymal state. However, β-catenin expression, as another marker of EMT, in the AGS-MSC treated group was not significantly different from the control group. Although increased expression of β-catenin is expected during the EMT process, due to the complex and extensive network of factors secreted by mesenchymal cells, there may be an interference with this process. It is believed that induction of EMT is correlated with phosphoinositide 3-kinase/Akt and Wnt/β-catenin signaling pathways (26). A number of studies have demonstrated that some secreted factors of MSCs inhibit Wnt and Akt signaling pathway in cancer cells. A study by Song et al. suggested that dickkopf-1 (Dkk-1), as one of the secreted factors of MSCs, inhibits Wnt signaling pathway in human MCF-7 breast cancer cells (27). Hence, failure to see the expected significant expression of β-catenin can occur due to other interfering secreted factors of MSCs.

Additionally, E-cadherin expression level was reduced significantly in the AGS cells co-cultured with MSCs. Epithelial cells are detectable by E-cadherin expression where the disappearance of this marker is a major sign of EMT occurrence (28). Taken together, co-culturing of AGS cells with MSCs can induce EMT and further promote tumor development.

Inconsistent with our study, a previous study by Hidehiko Takigawa et al. reported that the potential of the proliferation and migration of KM12SM colon cancer cells increased in direct co-culture with MSCs. They observed elevation in the expression of EMT-related genes including, fibronectin, SPARC, and galectin 1 following KM12SM- MSCs co-culture via microarray analysis. Furthermore, they performed qRT-PCR of EMT-related genes, which provided supportive evidence for microarray results. Hence, their results suggested that MSCs may have a great ability in inducing EMT in colon cancer cells causing colon cancer metastasis (29).

Another study by Sendurai A et al. revealed that MSCs secreted cytokines affected breast cancer cells by altering the expression of genes, including E-cadherin, Vimentin, Twist, and Snail. As mentioned above, these genes are involved in the modulation of epithelial state transition to mesenchymal state and thus developing breast cancer cells capable of migration and invasion (30).

In contrast with previous studies, Clarke et al. demonstrated that MSCs decreased the migration and invasion of human breast cancer cells via modulating TIMP-1 and -2 secretion (22).

Overall, in the current study, the induction of EMT was examined by qRT-PCR in three experimental groups (AGS cells without any treatment, AGS-MSC co-cultured group, and AGS treated with TGF-β group). Our results revealed that co-culturing AGS cells with MSCs results in overexpression of Snail and Vimentin as well as TGF-β treated group compared to AGS cells alone. Additionally, E-cadherin expression was significantly down-regulated. However, β-catenin expression, in AGS-MSC co-cultured group was not significantly altered in comparison with AGS cells without any treatment. Taken together, MSCs showed a high capacity to induce EMT in gastric cancer cell line.

## Conflict of interests

The authors declare that they have no conflict of interest.
